# Regional spread of an atypical ESBL-producing *Escherichia coli* ST131H89 clone among different human and environmental reservoirs in Western Switzerland

**DOI:** 10.1128/aac.00925-23

**Published:** 2024-01-03

**Authors:** Romain Martischang, Helena Seth-Smith, Tess D. Verschuuren, Delphine Héquet, Nadia Gaïa, Patrice François, Ad C. Fluit, Jan A. J. W. Kluytmans, Salome N. Seiffert, Evelina Tacconelli, Abdessalam Cherkaoui, Stephan Harbarth, Adrian Egli, Philipp Kohler

**Affiliations:** 1 Infection Control Programme and WHO Collaborating Centre, University of Geneva Hospitals and Faculty of Medicine, Geneva, Switzerland; 2 Clinical Bacteriology and Mycology, University Hospital Basel, Basel, Switzerland; 3 Department of Biomedicine, Applied Microbiology Research, Basel University, Basel, Switzerland; 4 Institute of Medical Microbiology, University of Zurich, Zürich, Switzerland; 5 Mahidol-Oxford Tropical Medicine Research Unit, Faculty of Tropical Medicine, Mahidol University, Bangkok, Thailand; 6 Unité Cantonale Hygiène, Prévention et Contrôle de l’infection, Canton de Vaud, Switzerland; 7 Genomic Research Laboratory, Geneva University Hospitals, Geneva, Switzerland; 8 Department of Medical Microbiology, University Medical Center Utrecht, Utrecht, the Netherlands; 9 Division of Human Microbiology, Centre for Laboratory Medicine, St. Gallen, Switzerland; 10 Department of Diagnostics and Public Health, Infectious Diseases, Verona University, Verona, Italy; 11 Department of Internal Medicine Infectious Diseases, Tübingen University, Tübingen, Germany; 12 Bacteriology Laboratory, Geneva University Hospitals, Geneva, Switzerland; 13 Division of Infectious Diseases and Hospital Epidemiology, Cantonal Hospital St Gallen, St Gallen, Switzerland; Shionogi Inc., Florham Park, New Jersey, USA

**Keywords:** *Escherichia coli*, molecular epidemiology, ST131, antimicrobial resistance, surveillance, long-term care facility

## Abstract

We describe the inter-regional spread of a novel ESBL-producing *Escherichia coli* subclone (ST131H89) in long-term care facility residents, general population, and environmental water sources in Western Switzerland between 2017 and 2020. The study highlights the importance of molecular surveillance for tracking emerging antibiotic-resistant pathogens in healthcare and community settings.

## INTRODUCTION

Over the last decades, ESBL-producing *Escherichia coli* (ESBL-EC) ST131 subclones carrying the *fimH*30 allele have spread worldwide, particularly in long-term care facilities (LTCFs) (1). Their frequent multidrug-resistant phenotype and potential for urinary tract and bloodstream infections warrant careful surveillance. Several recent studies have identified an atypical subclone ST131 *fimH*89 (ST131H89) in Swiss communities, LTCFs, and their surrounding environments (2–5). This manuscript integrates epidemiological and genomic data from three studies to estimate the prevalence of ST131H89, identify patient characteristics associated with ST131H89 carriage, and describe the genetic relatedness of identified strains. We also use genomic databases to interpret our findings in an international context.

## THE STUDY

This retrospective analysis synthetizes data from three previous observational studies. The first study performed annual prevalence surveys of ESBL-EC carriage between 2018 and 2020 among 2,403 residents of LTCFs in Western Switzerland (canton Geneva) (4). The second study was a cross-sectional analysis among 606 residents from 16 LTCFs in Western and Eastern Switzerland (cantons Vaud and St. Gallen) in 2019 (5). As part of an international research consortium (Understanding and modelling reservoirs, vehicles and transmission of ESBL-producing Enterobacteriaceae in the community and long-term care facilities MODERN) (6), we obtained microbiological data from a 2-year prospective international cohort of discharged patients with intestinal ESBL*-*EC carriage and their household contacts (2), LTCFs residents [only Spanish data published (7)], and environmental *E. coli* strains from Geneva (Western Switzerland) but also from non-Swiss sites including Sevilla (Spain), Tübingen (Germany), Utrecht (Netherlands), and Besançon (France), collected between 2017 and 2019 (3). Further information about study design, microbiological data, and processed samples is included in Table S2.

Using χ and Mann-Whitney U tests, we compared carriers of ST131H89 and other ST131 *E. coli* isolates regarding gender, age, country, recruitment center, sampling date and site, admission to an acute care institution in the last 6 months, endoscopy procedure in the last 6 months, and known prior ESBL-EC carriage. The prevalence of ST131H89 among all ST131 carriers was stratified by setting and country. We considered the first isolate per person and all environmental isolates, except for cluster analysis, where all isolates were included to prevent potential selection bias.

For microbiological analyses, we included all human and environmental ST131H89 isolates from the respective studies; for genomic analyses, we compared our isolates with previously characterized ST131H89 isolates obtained from published evidence (c.f. Appendix S1). All studies used phenotypic screening to detect ESBL-EC and next generation sequencing (NGS) as previously described (c.f. Appendix S1). Genome assemblies were generated using unicycler 0.3.b from which *fimH* types and *bla* genes were detected. Mutations in *gyrA* were detected from the assemblies using ResFinder v4.1 with 90% threshold for %ID and 60% minimum length and compared against SNP calls from CLC Genomics Workbench v20.0.2 (below) and core genome multi-locus sequence typing target genes. To generate an optimal reference genome, Oxford nanopore technology using R9.4 flowcell and rapid sequencing kit was used to sequence DNA extracted from isolate VD-05-035 (1950057932) (LTCF resident, Vaud) to mean 332 × coverage. The hybrid assembly was created using unicycler v0.4.8 resulting in contigs of 4970663 (circular), 69081 (circular), 58154, 5941, 5826, 2418, 462, and 373. Genomic relatedness was assessed within ESBL-EC ST131H89 isolates using a neighbor joining SNP tree created in CLC Genomic Workbench v20.0.2, with parameters that differed from the default as follows: variant calling with 10 × minimum coverage, 10 minimum count and 70% minimum frequency, and SNP tree creation with 10 × minimum coverage, 10% minimum coverage, 0 prune distance, and including multi-nucleotide variants. Belonging to a putatively relevant genomic cluster was defined as a pairwise distance of ≤10 SNP differences as described elsewhere (8).

Of the 207 ESBL-EC ST131 isolates included between October 2017 and February 2020, 126 originated from LTCF patients, 39 from discharged patients (*n* = 23) and community residents (*n* = 16), and 42 from different environmental water sources (LTCF outflow, wastewater treatment plants inflow, rivers, surfaces, U-bends). The ST131 isolates were predominantly from Switzerland with 97 (46.9%) and Spain with 55 (26.6%; Table 1). Among all ST131 isolates, ST131H89 (18.8%, *n* = 39) were observed only in Western Switzerland (Geneva and Vaud; Fig. S1). Among 76 Swiss LTCF residents, 12 Swiss community residents, and 9 Swiss environmental samples (single river), ST131H89 was respectively observed in 34 (44.7%), 3 (25.0%), and 2 (22.2%) samples (Table 1; Fig. S1). ST131H89 carriers were slightly older [85.0 (IQR 72.0–89.0) vs 73.0 years (IQR 62.0–86.0); *P* < 0.001] and were more likely to have had a prior endoscopy in the previous 6 months (*P* = 0.03) compared to ST131 carriers with other *fimH* alleles. We observed no statistically significant difference regarding gender, prior ESBL carriage and hospitalization.

**TABLE 1:** Proportion of ST131H89 among all ST131 strains

Location		ESBL-EC ST131 (*n* = 207)	ESBL-EC ST131 non-H89 (*n* = 168)	ESBL-EC ST131H89 (*n* = 39)	*P*-value
Switzerland, Vaud	Patient number (%) among 8 LTCFs	29 (100%)	17 (58.6%)	12 (41.4%)	
Switzerland, St.Gallen	Patient number (%) among 8 LTCFs	13 (100%)	13 (100%)	0 (0.0 %)	
Switzerland, Geneva	Patient number among 1 LTCF (%)	34 (100%)	12 (35.3%)	22 (64.7%)	
	Number of community residents (%)	12 (100%)	9 (75%)	3 (25%)	
	Number of environmental isolates (%)	9 (100%)	7 (77.8%)	2 (22.2%)	
Netherlands, Germany, France, Spain	Patient number among LTCF (%)	50 (100%)	50 (100%)	0 (0.0 %)	
	Number of community residents (%)	27 (100%)	27 (100%)	0 (0.0 %)	
	Number of environmental isolates (%)	33 (100%)	33 (100%)	0 (0.0 %)	
Participant characteristics (*n* = 165 LTCF and community residents)					
Age (median, IQR)		77 (IQR 64.0–87.8)	73.0 (IQR 62.0–86.0)	85 (IQR 72.0–89.0)	<0.001
Gender (female, %)		55 (100%)	37 (67.3%)	18 (32.7%)	1
Prior endoscopy (%)^*a*^		6 (100%)	0 (0.0%)	6 (100%)	0.03
Prior hospitalization (%)		67 (100%)	42 (62.7%)	25 (37.3%)	0.23
Known carrier (%)		15 (100%)	10 (66.7%)	5 (33.3%)	0.38

^*a*^ Data available for 77 patients.

The eight contigs comprising the reference isolate genome included a circularized chromosome of 5 Mb, a circularized plasmid of 69 Kb, and other contigs representing further plasmids. A total of 55 ST131H89 isolates (c.f. Table S2) underwent NGS. All the ST131H89 strains carried *bla*_CTX-M-14_ (chromosomal) and *bla*_TEM-1_ (plasmid borne). All were also found to possess a *gyrA* mutation associated with quinolone resistance (S83L).

**Fig 1:**
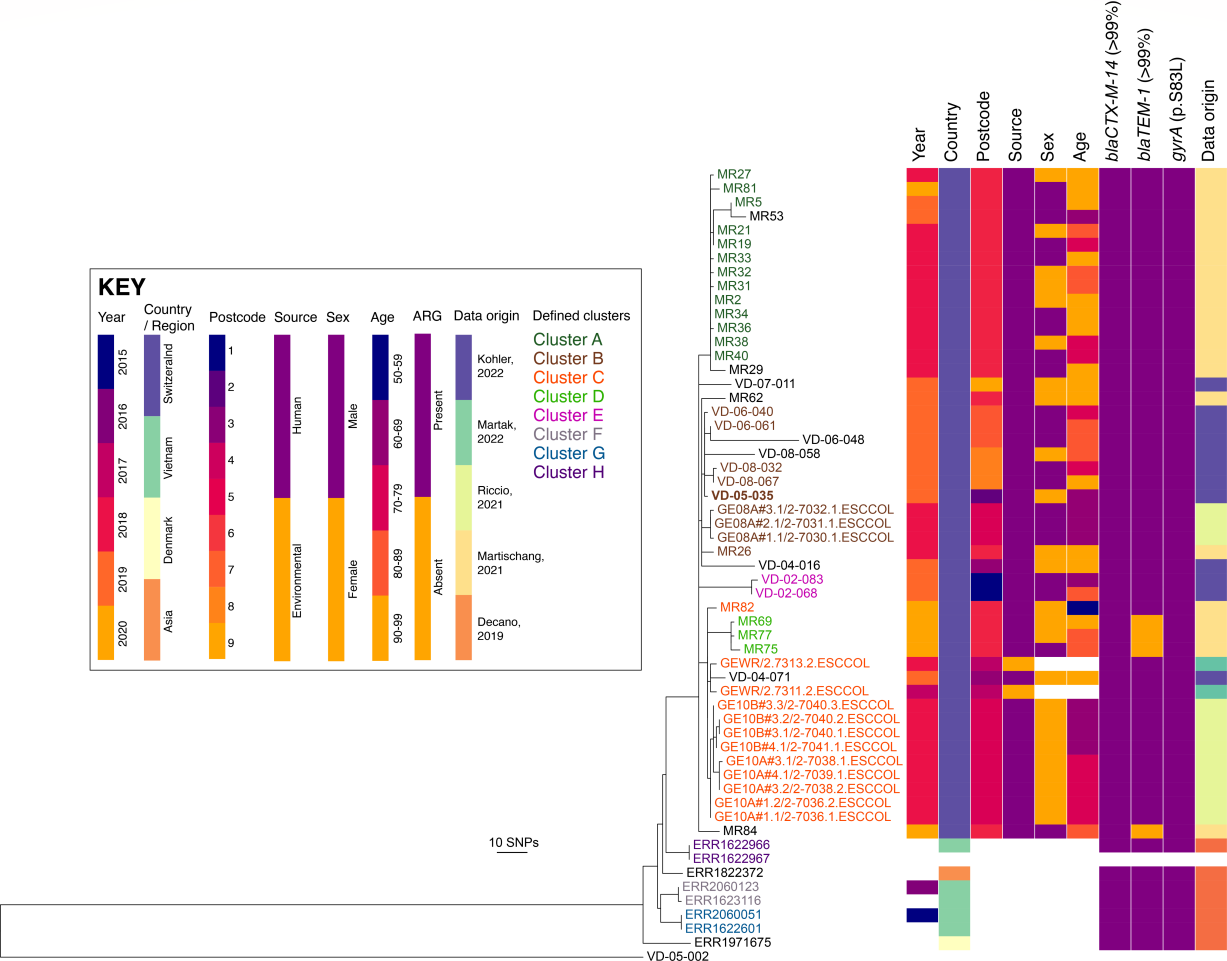
Annotated neighbour joining SNP tree of ST131H89. Reads were mapped against a hybrid assembled reference of isolate VD-05-035 (1950057932) in CLC Genomics Workbench (see methods). Postal codes are anonymized as 1–10, defining geographical regions within Switzerland. ARG defines the presence of resistance genes. Isolates n° GE08A, GE10A and GE10B originated from three unique participants in two households (GE08 and GE10), with four follow-ups (e.g., GE08#1–4) and up to four colonies analyzed per sample (e.g., GE08#1.1–4).

In terms of clonal relatedness, we identified by cgMLST 13 potential transmission clusters among ST131 isolates, defined by a 10-allele difference cut-off, including a multicentric cluster of 56 ST131H89 isolates (Fig. S2). Using further discriminatory analysis such as the annotated neighbor joining SNP tree and the SNP matrix, relationships between isolates from different locations could be seen in eight clusters (A–H) (Fig. 1; Table S3). Cluster A included 13 residents (0–10 SNPs) sampled in January 2018 from a single Geneva LTCF, most of the patients were elderly male, hospitalized in the past 6 months. Cluster B included nine isolates (0–9 SNPs), from five residents of three LTCFs in Vaud, one community resident and one LTCF resident in Geneva, living <5 km apart, and sampled between January and May 2018. Both Geneva residents had been hospitalized in the past 6 months. All Vaud residents were sampled 1 year later in 2019. To note, the three isolates from one household resident (GE08A) were collected within the visits #1, #2, and #3, respectively, at baseline, 1 week, and 2 months. Cluster C included 12 Geneva strains (0–8 SNPs) from two communities and one LTCF resident and two isolates from a river sample (Rhône). The environmental isolates were sampled in December 2017 and February 2018, with <2 km from the Geneva LTCF where a clonally related isolate was identified 2 years later. Both community residents were sampled <6 km in June 2018. The five isolates from one household resident (GE10A) represent five colonies analyzed from three sampling dates, within the visits #1, #3, and #4, respectively, at baseline, 1 week, and 4 months. The four isolates from one resident from the same prior household (GE10B) represent four colonies analyzed from two sampling dates, within the visits #3 and #4, respectively, at 2 and 4 months. Cluster D included three strains (2–4 SNP) from three residents of the same Geneva LTCF, sampled in January 2020. Cluster E included two strains (2 SNP) from two residents of the same Vaud LTCF, sampled in 2019. The three remaining clusters (F, G, and H) each included two isolates, sampled from Vietnam, without much epidemiological information. Swiss ST131H89 strains differed from international strains by over 22 SNPs.

## CONCLUSION

Among a collection of ST131 ESBL-EC from different countries and settings, we observed an inter-regional cluster of clonally related ST131H89 isolates carrying *bla*_CTX-M-14_ and *bla*_TEM-1_ beta-lactamases in Western Switzerland, mainly among LTCF residents but also in samples of community residents and environmental water. Both clusters B and C were of particular interest, observing clonally related ST131H89 isolates across multiple cantons, with spatio-temporal links observed for two patients in Geneva. The second cluster represents a geographic cluster between LTCF residents and a river sample, 2 years apart. To date, ESBL-EC ST131H89 has only been sporadically identified in various countries, in human, and environmental isolates (hospital sewage, inflow to WWTP) (9, 10) but no clonal outbreak has been reported (11). In contrast to the hyperendemic ST131H30, isolates carrying H89 *fimH* type do not exhibit a multi-resistant phenotype, with most isolates being phenotypically susceptible to quinolones and compounds commonly used to treat urinary tract infections such as nitrofurantoin or fosfomycin. Nevertheless, the regional spread of a single H89 clone with no apparent epidemiological link between different reservoirs, including the aquatic environment, suggests larger undetected transmission chains. Of concern, O16:H5-ST131/*fim*H41, which is a subclone closely related to ST131H89, has been reported from a neighboring country (southern Germany) harboring a *bla*_OXA-244_ carbapenemase gene (12). This example highlights the importance of close monitoring of ESBL-EC clones of concern, especially considering the under-detection issues related to harboring (13) and the lack of genotypic confirmation of carbapenemase-producing Enterobacterales in certain countries (14). Limitations of our study include a sampling bias toward LTCFs residents and Western Switzerland, which prevents us from assessing the prevalence of ST131 H89 in a broader population. Second, the prevalence of this novel clone was assessed in ST131 strains and not in all participants or ESBL-EC carriers, limiting potential interpretations considering its impact on ST131 or ESBL-EC prevalence. Third, definition of clonal relatedness used a threshold of 10 SNPs; use of a higher or lower threshold would slightly alter the interpretation of the data. Effectively, MR29 was <10 SNP away from all strains of cluster A except one (12 SNP from MR5). Thus, in the absence of clear epidemiological links between individuals, we cannot ascertain cross-transmission events (8, 15). In conclusion, intensified molecular surveillance programs are needed on single strain levels to better monitor the spread of ESBL-EC ST131H89 and to identify potential reservoirs that may allow the implementation of targeted containment measures.

## Data Availability

The datasets used and/or analyzed is detailed in the appendix. Any further information is available from the corresponding author on reasonable request.

## SUPPLEMENTAL MATERIAL

Supplemental material aac.00925-23-s0001.docx Appendix S1, Tables S1 to S3, and Fig. S1 and S2.

ASM does not own the copyrights to Supplemental Material that may be linked to, or accessed through, an article. The authors have granted ASM a non-exclusive, world-wide license to publish the Supplemental Material files. Please contact the corresponding author directly for reuse.
