# Supplementary material for: Regional spread of an atypical ESBL-producing Escherichia coli ST131H89 clone among different human and environmental reservoirs in Western Switzerland
Source: Antimicrob Agents Chemother. 2024 Jan 3;68(2):e00925-23. doi: 10.1128/aac.00925-23 (PMC10848748; doi:10.1128/aac.00925-23)
Supplement: Supplemental material — Appendix S1, Tables S1 to S3, and Fig. S1 and S2. [file aac.00925-23-s0001.docx]

**Supplementary Online Content**

**Regional spread of an atypical ESBL-producing *Escherichia coli*** **ST131H89 clone among different human and environmental reservoirs in Western Switzerland**

Romain Martischang^1^, Helena Seth-Smith^2,3,4^, Tess D. Verschuuren^5^, Delphine Héquet^6^, Nadia Gaïa^7^, Patrice François^7^, Ad C. Fluit^8^, Jan A.J.W. Kluytmans^8^, Salome N. Seiffert^9^, Evelina Tacconelli^10^, Abdessalam Cherkaoui^11^, Stephan Harbarth^1^, Adrian Egli^2,3,4^, Philipp Kohler^12^

^1^ Infection Control Programme and WHO Collaborating Centre, University of Geneva Hospitals and Faculty of Medicine, Geneva, Switzerland

^2^ Clinical Bacteriology and Mycology, University Hospital Basel, Basel, Switzerland

^3^ Applied Microbiology Research, Department of Biomedicine, Basel University, Basel, Switzerland

^4^ Institute of Medical Microbiology, University of Zurich, Zürich, Switzerland

^5^ Mahidol-Oxford Tropical Medicine Research Unit, Faculty of Tropical Medicine, Mahidol University, Bangkok, Thailand

^6^ Unité cantonale hygiène, prévention et contrôle de l’infection, Canton de Vaud, Switzerland

^7^ Genomic Research Laboratory, Geneva University Hospitals, Geneva, Switzerland.

^8^ Department of Medical Microbiology, University medical Center Utrecht, Utrecht, the Netherlands

^9^ Division of Human Microbiology, Centre for Laboratory Medicine, St. Gallen, Switzerland

^10^ Infectious Diseases, Department of Diagnostics and Public Health, Verona University, Verona, Italy; Infectious Diseases, Department of Internal Medicine I, Tübingen University, Tübingen, Germany

^11^ Bacteriology Laboratory, Geneva University Hospitals, Geneva, Switzerland.

^12^ Cantonal Hospital St Gallen, Division of Infectious Diseases and Hospital Epidemiology, St Gallen, Switzerland

**Appendix S1. Microbiological methods**

For microbiological analyses, we included all human and environmental ST131H89 isolates from the respective studies; for genomic analyses, we compared our isolates with previously characterized ST131H89 isolates obtained from published evidence (1). For more information, the origin and availability of all strains used in this study is detailed in eTable 1.

All studies used phenotypic screening to detect ESBL-EC as previously described (2–5). Next generation sequencing (NGS) was performed as previously described (2–5). Genome assemblies were generated using unicycler 0.3.b (6) from which *fimH* types (7) and *bla* genes (8,9) were detected. Mutations in *gyrA* were detected from the assemblies using ResFinder v4.1 with 90% threshold for %ID and 60% minimum length (10–12) and compared against SNP calls from CLC Genomics Workbench v20.0.2 (below) and core genome multi-locus sequence typing (cgMLST) target genes. To generate an optimal reference genome, Oxford nanopore technology using R9.4 flowcell and rapid sequencing kit was used to sequence DNA extracted from isolate VD-05-035 (1950057932) (LTCF resident, Vaud) to mean 332x coverage. The hybrid assembly was created using unicycler v0.4.8 (6) resulting in contigs of 4970663 (circular), 69081 (circular), 58154, 5941, 5826, 2418, 462 and 373 bases pairs. Genomic relatedness was assessed within ESBL-EC ST131H89 isolates using a neighbor joining SNP tree created in CLC Genomic Workbench v20.0.2, with parameters that differed from the default as follows: variant calling with 10x minimum coverage, 10 minimum count and 70% minimum frequency, and SNP tree creation with 10x minimum coverage, 10% minimum coverage, 0 prune distance and including multi-nucleotide variants (MNVs). Belonging to a putatively relevant genomic cluster was defined as a pairwise distance of ≤10 SNP differences as described elsewhere (13).

**Table S1. Origin and availability of strains**

| **Sample** | **Alternative ID** | **Sample Accession** | **Assemblies submitted** | **Project** | **Year** | **Country** | **Data origin** |
| --- | --- | --- | --- | --- | --- | --- | --- |
| 1950036951-19 | VD-02-068 | ERS13469312 | yes | PRJEB56148 | 2019 | Switzerland | Kohler 2022 |
| 1950037716-19 | VD-02-083 | ERS13469313 | yes | PRJEB56148 | 2019 | Switzerland | Kohler 2022 |
| 1950057932 | VD-05-035 | ERS13469326 | yes | PRJEB56148 | 2019 | Switzerland | Kohler 2022 |
| 1950046292 | VD-04-016 | ERS13469318 | yes | PRJEB56148 | 2019 | Switzerland | Kohler 2022 |
| 1950047105 | VD-04-071 | ERS13469321 | yes | PRJEB56148 | 2019 | Switzerland | Kohler 2022 |
| 27311_2_ESCCOL_S40_HQ_1 |  | *data not public yet* | no | PRJEB50545 | 2017 | Switzerland | Martak 2022 |
| 27313_2_ESCCOL_S44_HQ_1 |  | *data not public yet* | no | PRJEB50545 | 2018 | Switzerland | Martak 2022 |
| ECO-JSC-MOD-104312 |  | *data not public yet* | no | PRJEB50545 | 2018 | Switzerland | Riccio 2021 |
| ECO-JSC-MOD-104313 |  | *data not public yet* | no | PRJEB50545 | 2018 | Switzerland | Riccio 2021 |
| ECO-JSC-MOD-104314 |  | *data not public yet* | no | PRJEB50545 | 2018 | Switzerland | Riccio 2021 |
| ECO-JSC-MOD-104318 |  | *data not public yet* | no | PRJEB50545 | 2018 | Switzerland | Riccio 2021 |
| ECO-JSC-MOD-104642 |  | *data not public yet* | no | PRJEB50545 | 2018 | Switzerland | Riccio 2021 |
| ECO-JSC-MOD-104319 |  | *data not public yet* | no | PRJEB50545 | 2018 | Switzerland | Riccio 2021 |
| ECO-JSC-MOD-104643 |  | *data not public yet* | no | PRJEB50545 | 2018 | Switzerland | Riccio 2021 |
| ECO-JSC-MOD-104320 |  | *data not public yet* | no | PRJEB50545 | 2018 | Switzerland | Riccio 2021 |
| ECO-JSC-MOD-104321 |  | *data not public yet* | no | PRJEB50545 | 2018 | Switzerland | Riccio 2021 |
| ECO-JSC-MOD-104644 |  | *data not public yet* | no | PRJEB50545 | 2018 | Switzerland | Riccio 2021 |
| ECO-JSC-MOD-104645 |  | *data not public yet* | no | PRJEB50545 | 2018 | Switzerland | Riccio 2021 |
| ECO-JSC-MOD-104322 |  | *data not public yet* | no | PRJEB50545 | 2018 | Switzerland | Riccio 2021 |
| MR19 |  | MR19 | no | PRJEB68082 | 2018 | Switzerland | Martischang 2021 |
| MR2 |  | MR2 | no | PRJEB68082 | 2018 | Switzerland | Martischang 2021 |
| MR5 |  | MR5 | no | PRJEB68082 | 2019 | Switzerland | Martischang 2021 |
| MR21 |  | MR21 | no | PRJEB68082 | 2018 | Switzerland | Martischang 2021 |
| MR26 |  | MR26 | no | PRJEB68082 | 2018 | Switzerland | Martischang 2021 |
| MR27 |  | MR27 | no | PRJEB68082 | 2018 | Switzerland | Martischang 2021 |
| MR29 |  | MR29 | no | PRJEB68082 | 2018 | Switzerland | Martischang 2021 |
| MR31 |  | MR31 | no | PRJEB68082 | 2018 | Switzerland | Martischang 2021 |
| MR32 |  | MR32 | no | PRJEB68082 | 2018 | Switzerland | Martischang 2021 |
| MR33 |  | MR33 | no | PRJEB68082 | 2018 | Switzerland | Martischang 2021 |
| MR34 |  | MR34 | no | PRJEB68082 | 2018 | Switzerland | Martischang 2021 |
| MR36 |  | MR36 | no | PRJEB68082 | 2018 | Switzerland | Martischang 2021 |
| MR38 |  | MR38 | no | PRJEB68082 | 2018 | Switzerland | Martischang 2021 |
| MR40 |  | MR40 | no | PRJEB68082 | 2018 | Switzerland | Martischang 2021 |
| MR53 |  | MR53 | no | PRJEB68082 | 2019 | Switzerland | Martischang 2021 |
| MR62 |  | MR62 | no | PRJEB68082 | 2019 | Switzerland | Martischang 2021 |
| MR69 |  | MR69 | no | PRJEB68082 | 2020 | Switzerland | Martischang 2021 |
| MR75 |  | MR75 | no | PRJEB68082 | 2020 | Switzerland | Martischang 2021 |
| MR77 |  | MR77 | no | PRJEB68082 | 2020 | Switzerland | Martischang 2021 |
| MR81 |  | MR81 | no | PRJEB68082 | 2020 | Switzerland | Martischang 2021 |
| MR82 |  | MR82 | no | PRJEB68082 | 2020 | Switzerland | Martischang 2021 |
| MR84 |  | MR84 | no | PRJEB68082 | 2020 | Switzerland | Martischang 2021 |
| 1950064778-19 | VD-06-048 | ERS13469332 | yes | PRJEB56148 | 2019 | Switzerland | Kohler 2022 |
| 1950064792-19 | VD-06-061 | ERS13469333 | yes | PRJEB56148 | 2019 | Switzerland | Kohler 2022 |
| 1950064798-19 | VD-06-040 | ERS13469331 | yes | PRJEB56148 | 2019 | Switzerland | Kohler 2022 |
| 1950061883 | VD-08-067 | ERS13469340 | yes | PRJEB56148 | 2019 | Switzerland | Kohler 2022 |
| 1950061888 | VD-08-058 | ERS13469339 | yes | PRJEB56148 | 2019 | Switzerland | Kohler 2022 |
| 1950061894 | VD-08-032 | ERS13469338 | yes | PRJEB56148 | 2019 | Switzerland | Kohler 2022 |
| 1950044155-19 | VD-07-011 | ERS13469334 | yes | PRJEB56148 | 2019 | Switzerland | Kohler 2022 |
| ERR1622966 | 78006a50-fa7c-11e5-b5da-3c4a9275d6c8 | ERS1153146 | no | PRJEB12887 | unknown | Vietnam | Decano 2019 |
| ERR1622601 | 29faf730-fa7c-11e5-8812-3c4a9275d6c8 | ERS1152385 | no | PRJEB12887 | unknown | Vietnam | Decano 2019 |
| ERR2060051 | CT30F.G1 | ERS1847717 | no | PRJEB21997 | 2015 | Vietnam | Decano 2019 |
| ERR1623116 | 75ce6f20-fa7c-11e5-9ab9-3c4a9275d6c8 | ERS1153093 | no | PRJEB12887 | unknown | Vietnam | Decano 2019 |
| ERR2060123 | MT37R.N1 | ERS1847789 | no | PRJEB21997 | 2016 | Vietnam | Decano 2019 |
| ERR1971675 | Q1WOLBK0 | ERS1724525 | no | PRJEB20792 | unknown | Denmark | Decano 2019 |
| ERR1822372 | 5b911d50-7fd4-11e6-b8fe-3c4a9275d6c8 | ERS1369186 | no | PRJEB15430 | unknown | Asia | Decano 2019 |
| ERR1622967 | 78403270-fa7c-11e5-b5da-3c4a9275d6c8 | unknown | no | PRJEB12887 | unknown | Vietnam | Decano 2019 |

**Table S2. Study design, microbiological methods and processed samples from the different studies**

| **Study** | **Riccio ME et al. *Clin Microbiol Infect*. 2021 (MODERN project)** (2) **^a^** | **Martak D. *Clin Microbiol Infect Dis.* 2021**  **(MODERN project)** (5) **^a^** | **Salamanca-Rivera, *Microbiology spectrum.* 2022 (MODERN project)** (14) **^a, b^** | | **Martischang R. *Antimicrob Resist Infect Control.* 2021** (3) | **Kohler P. *J Am Med Dir Assoc*. 2021** (4) |
| --- | --- | --- | --- | --- | --- | --- |
| **Study design** | | | | | | |
| **Date** | November 2017 – April 2019 | January 2018 – August 2019 | March – April 2018 and  November 2018 to January 2019 | January 2018 – February 2020 | | August – October 2019 |
| **Countries** | Switzerland  Spain  Germany  Netherlands  France | Switzerland  Spain  Germany  Netherlands  France | Spain  Germany  Netherlands  France | Switzerland | | Switzerland |
| **Participants** | Index cases known as intestinal ESBL-PE carriers and household contacts | Environmental analysis | Long-term care facility residents | Long-term care facility residents | | Long-term care facility residents |
| **Sampling scheme** | Baseline visit, follow-up at 1 week, 2 months, 4 months | 8 times over a 32-week period | Baseline, follow-up at weeks 1, 4, and 12. | Serial yearly cross-sectional samplings of all residents | | Cross-sectional samplings of all residents |
| **Sampling site** | Stool samples or rectal swabs. Rectal swabs were verified visually by the presence of faecal material. | 250mL and 500mL of water collected from (1) inflow of the downstream WWTP, LTCF discharge sewer samples and (2) from river samples 200m downstream of the WWTP outflow and >5m from the riverbank, processed within 4 hours of collection | Stool samples or rectal swabs | Stool samples or rectal swabs | | Rectal (all), urine (in case of urinary catheter), or wound (if applicable) screening |
| **Target pathogens** | ESBL-producing *E.coli* and *K.pneumoniae* | ESBL-producing *E.coli* and *K.pneumoniae* | Quinolone resistant *E.coli* | ESBL-producing *E.coli* | | ESBL-producing Enterobacterales |
| **Microbiological methods** | | | | | | |
| **Broth** | Yes | No | Yes | No | | Yes |
| **Media** | ChromID ESBL agar (bioMérieux, Marcy l’Etoile, France) and McConkey broth supplemented with vancomycin and cefuroxime. | ESBL specific plates | UTI agar (Oxoid®) with 2 mg/L ciprofloxacin | ChromID ESBL agar (bioMérieux, Marcy l’Etoile, France) | | ChromID ESBL agar (bioMérieux, Marcy l’Etoile, France) |
| **Speciation** | MALDI-TOF | MALDI-TOF | Not specified, possibly using chromogenic characteristics of the media | | MALDI-TOF | MALDI-TOF |
| **Resistance profiling** | DDST20, DDST30, and ESBL+AmpC Screen ID Kit (Rosco Diagnostica, Taastrup, Denmark) | DDST20, DDST30 | NMDRM1 Microscan panels (Beckam Coulter) | | DDST20, DDST30, and ESBL+AmpC Screen ID Kit (Rosco Diagnostica, Taastrup, Denmark) | BD Phoenix M50 (Becton Dickinson, Sparks, MD) with E-test ESBL confirmation using specific E-test stripes (bioMérieux, Marcy l’Etoile, France) |
| **Genotypic methods** | | | | | | |
| **Colonies analyzed per morphotype** | 1-4 morphotypes per sample | Maximum 3 morphotypes per samples | Not specified | | One morphotype per sample | Not specified |
| **Extraction** | QIAGEN DNeasy UltraClean Microbial Kit (Qiagen Hilden, Germany) | QIAGEN DNeasy UltraClean Microbial Kit (Qiagen Hilden, Germany) | Not specified | | QIAGEN DNeasy UltraClean Microbial Kit (Qiagen Hilden, Germany) | EZ1, Qiacube robotic system (Qiagen, Hilden, Germany) |
| **Library preparation** | Not specified | Nextera DNA Flex Library Prep Kit (Illumina, San Diego, CA, USA) | Nextera DNA Flex Library Prep Kit (Illumina, San Diego, CA, USA) | | Nextera DNA Flex Library Prep Kit (Illumina, San Diego, CA, USA) | Nexteraflex (Illumina, San Diego, CA) |
| **Instrument** | Illumina NextSeq | Illumina NextSeq | Illumina MiSeq | | Illumina HiSeq | Illumina NextSeq |
| **Results** | | | | | | |
| **Number of ESBL-producing *E.coli* per unique patient** | 121 (121 ESBL-*E.coli* sequenced) | 158 (158 ESBL-*E.coli* sequenced) | Not specified **^b^** | | 93 | 62 (59 ESBL-*E.coli* sequenced) |
| **Number of ESBL-producing ST131 *E.coli* per unique patient** | Not specified (per isolate and not per patient) | 42 ^c^ | Not specified **^b^** | | 54 (34 ESBL -*E.coli* ST131 sequenced) | 43 |
| **Number of ESBL-producing ST131 *E.coli* included in our study per unique patient (total isolate number)** | 39 (306) | 42 ^c^ (42) | 50 (184) | | 34 (34) | 43 (43) |
| **Number of ESBL-producing ST131H89 *E.coli* per unique patient (total isolate number)** | 3 (12) | 2 (2) | 0 (0) | | 22 (22) | 12 (12) |

^a^ MODERN : Multicentre project called “Understanding and modelling reservoirs, vehicles and transmission of ESBL-producing Enterobacteriaceae in the community and long term care facilities”

^b^ Screening of LTCF residents has been performed as a part of MODERN project (Work Package 1) in Besançon (France), Sevilla (Spain), Tubingen (Germany), and Utrecht (Netherlands), with 9 isolates from 2 patients, 118 isolates from 34 patients, 52 isolates from 11 patients and 5 isolates from 3 patients. Only results from 2 LTCFs in Sevilla were available online (82 residents, with 42 colonized by FQ-R ST131 *E.coli*)

^c^ U-bends and surfaces are included (in the original articles, n=34 ESBL-EC ST131 in the aquatic environment). Multiple isolates per environmental sample were considered.

**Table S3. Epidemiological information on ESBL-producing *Escherichia coli* ST131 H89 samples (n=56)**

| ID | Cluster | Postcode | Sex | Age | Prior hospitalization | Prior endoscopy | Prior carriage | Sampling date | Sampling site | Species | source |
| --- | --- | --- | --- | --- | --- | --- | --- | --- | --- | --- | --- |
| MR2 | A | 1233 | f | 98 | yes | no | 16.12.2017 | 09.01.2018 | rectal | *E. coli* | LTCF |
| MR5 | A | 1233 | m | 91 | no | no | 12.10.2018 | 08.01.2019 | rectal | *E. coli* | LTCF |
| MR21 | A | 1233 | f | 83 | yes | no |  | 08.01.2018 | rectal | *E. coli* | LTCF |
| MR27 | A | 1233 | f | 93 | yes | no | 18.04.2012 | 22.01.2018 | rectal | *E. coli* | LTCF |
| MR31 | A | 1233 | f | 87 | yes | no | 21.10.2017 | 23.01.2018 | rectal | *E. coli* | LTCF |
| MR33 | A | 1233 | m | 95 | yes | no |  | 23.01.2018 | rectal | *E. coli* | LTCF |
| MR38 | A | 1233 | f | 71 | no | no |  | 24.01.2018 | rectal | *E. coli* | LTCF |
| MR40 | A | 1233 | m | 77 | no | no |  | 25.01.2018 | rectal | *E. coli* | LTCF |
| MR19 | A | 1233 | m | 74 | yes | yes |  | 08.01.2018 | rectal | *E. coli* | LTCF |
| MR32 | A | 1233 | f | 86 | yes | yes |  | 23.01.2018 | rectal | *E. coli* | LTCF |
| MR34 | A | 1233 | m | 94 | yes | yes | 25.02.2017 | 23.01.2018 | rectal | *E. coli* | LTCF |
| MR36 | A | 1233 | m | 95 | yes | yes |  | 24.01.2018 | rectal | *E. coli* | LTCF |
| MR81 | A | 1233 | m | 90 | yes | yes | 06.12.2019 | 24.01.2020 | rectal | *E. coli* | LTCF |
| VD-05-035 | B | 1010 | f | 63 | no | no |  | 02.10.2019 | rectal | *E. coli* | LTCF |
| VD-06-040 | B | 1400 | m | 73 | no | no |  | 02.10.2019 | rectal | *E. coli* | LTCF |
| VD-06-061 | B | 1400 | m | 88 | no | no |  | 02.09.2019 | rectal | *E. coli* | LTCF |
| VD-08-032 | B | 1450 | m | 79 | no | no |  | 29.09.2019 | rectal | *E. coli* | LTCF |
| VD-08-067 | B | 1450 | m | 90 | no | no |  | 29.09.2019 | rectal | *E. coli* | LTCF |
| MR26 | B | 1233 | f | 90 | yes | no |  | 22.01.2018 | rectal | *E. coli* | LTCF |
| GE08A#1.1/2-7030.1.ESCCOL ^a^ | B | 1214 | m | 60 | yes |  |  | 06.05.2018 | stools | *E. coli* | Community |
| GE08A#2.1/2-7031.1.ESCCOL ^a^ | B | 1214 | m | 60 | yes |  |  | 14.05.2018 | stools | *E. coli* | Community |
| GE08A#3.1/2-7032.1.ESCCOL ^a^ | B | 1214 | m | 60 | yes |  |  | 02.07.2018 | stools | *E. coli* | Community |
| MR82 | C | 1233 | f | 52 | yes | no | 14.03.2019 | 28.01.2020 | rectal | *E. coli* | LTCF |
| GE10A#1.1/2-7036.1.ESCCOL ^a^ | C | 1217 | f | 71 | yes |  |  | 21.06.2018 | stools | *E. coli* | Community |
| GE10A#1.2/2-7036.2.ESCCOL ^a^ | C | 1217 | f | 71 | yes |  |  | 21.06.2018 | stools | *E. coli* | Community |
| GE10A#3.1/2-7038.1.ESCCOL ^a^ | C | 1217 | f | 71 | yes |  |  | 27.08.2018 | stools | *E. coli* | Community |
| GE10A#3.2/2-7038.2.ESCCOL ^a^ | C | 1217 | f | 71 | yes |  |  | 27.08.2018 | stools | *E. coli* | Community |
| GE10A#4.1/2-7039.1.ESCCOL ^a^ | C | 1217 | f | 71 | yes |  |  | 28.08.2018 | stools | *E. coli* | Community |
| GE10B#3.1/2-7040.1.ESCCOL ^a^ | C | 1217 | f | 68 | yes |  |  | 27.08.2018 | stools | *E. coli* | Community |
| GE10B#3.2/2-7040.2.ESCCOL ^a^ | C | 1217 | f | 68 | yes |  |  | 27.08.2018 | stools | *E. coli* | Community |
| GE10B#3.3/2-7040.3.ESCCOL ^a^ | C | 1217 | f | 68 | yes |  |  | 27.08.2018 | stools | *E. coli* | Community |
| GE10B#4.1/2-7041.1.ESCCOL ^a^ | C | 1217 | f | 68 | yes |  |  | 28.08.2018 | stools | *E. coli* | Community |
| GEWR/2.7311.2.ESCCOL | C | 1209 |  |  |  |  |  | 19.12.2017 | environmental | *E. coli* | River |
| GEWR/2.7313.2.ESCCOL | C | 1209 |  |  |  |  |  | 14.02.2018 | environmental | *E. coli* | River |
| MR69 | D | 1233 | f | 96 | no | no |  | 14.01.2020 | rectal | *E. coli* | LTCF |
| MR77 | D | 1233 | f | 88 | yes | no |  | 22.01.2020 | rectal | *E. coli* | LTCF |
| MR75 | D | 1233 | m | 80 | yes | yes |  | 20.01.2020 | rectal | *E. coli* | LTCF |
| VD-02-068 | E | 1004 | m | 84 | no | no |  | 21.08.2019 | rectal | *E. coli* | LTCF |
| VD-02-083 | E | 1004 | m | 68 | yes (CHUV Apr 30th to May 8th 2019) | no | 10.12.2015 | 21.08.2019 | urine | *E. coli* | LTCF |
| ERR1623116 | F | Vietnam |  |  |  |  |  | unknown |  | *E. coli* | Downing et al |
| ERR2060123 | F | Vietnam |  |  |  |  |  | 2016 |  | *E. coli* | Downing et al |
| ERR1622601 | G | Vietnam |  |  |  |  |  | unknown |  | *E. coli* | Downing et al |
| ERR2060051 | G | Vietnam |  |  |  |  |  | 2015 |  | *E. coli* | Downing et al |
| ERR1622966 | H | Vietnam |  |  |  |  |  | unknown |  | *E. coli* | Downing et al |
| ERR1622967 | H | unknown |  |  |  |  |  | unknown |  | *E. coli* | Downing et al |
| VD-04-016 |  | 1052 | f | 93 | no | no |  | 05.09.2019 | rectal | *E. coli* | LTCF |
| VD-04-071 |  | 1052 | f | 99 | no | no | 14.03.2018 | 05.09.2019 | rectal | *E. coli* | LTCF |
| VD-06-048 |  | 1400 | m | 87 | no | no |  | 02.10.2019 | rectal | *E. coli* | LTCF |
| VD-07-011 |  | 1530 | f | 94 | no | no |  | 24.09.2019 | rectal | *E. coli* | LTCF |
| VD-08-058 |  | 1450 | f | 85 | no | no |  | 29.09.2019 | rectal | *E. coli* | LTCF |
| MR29 |  | 1233 | m | 99 | yes | no |  | 22.01.2018 | rectal | *E. coli* | LTCF |
| MR53 |  | 1233 | m | 68 | yes | no |  | 08.01.2019 | rectal | *E. coli* | LTCF |
| MR62 |  | 1233 | f | 93 | no | no | 25.01.2019 | 26.01.2019 | rectal | *E. coli* | LTCF |
| MR84 |  | 1233 | m | 83 | no | no | 28.11.2019 | 07.01.2020 | rectal | *E. coli* | LTCF |
| ERR1971675 |  | Denmark |  |  |  |  |  | unknown |  | *E. coli* | Downing et al |
| ERR1822372 |  | Asia |  |  |  |  |  | unknown |  | *E. coli* | Downing et al |

^a^  Isolates n° GE08A, GE10A and GE10B originated from three unique participants in two households (GE08 and GE10), with 4 follow-ups (#1-4) and up to 4 colonies analyzed per sample (#1.1-4). Thus, we obtained seven colonies or strains coming from 3 samples (1-4 colonies analyzed per isolate). Three strains were possibly duplicated into 7 isolates.

**Figure S1. Regional distribution of ST131H89 strains in Switzerland among the different reservoirs. (The** map was generated using the leaflet package within the R software environment, incorporating data from OpenStreetMap [(<https://www.openstreetmap.org>].)


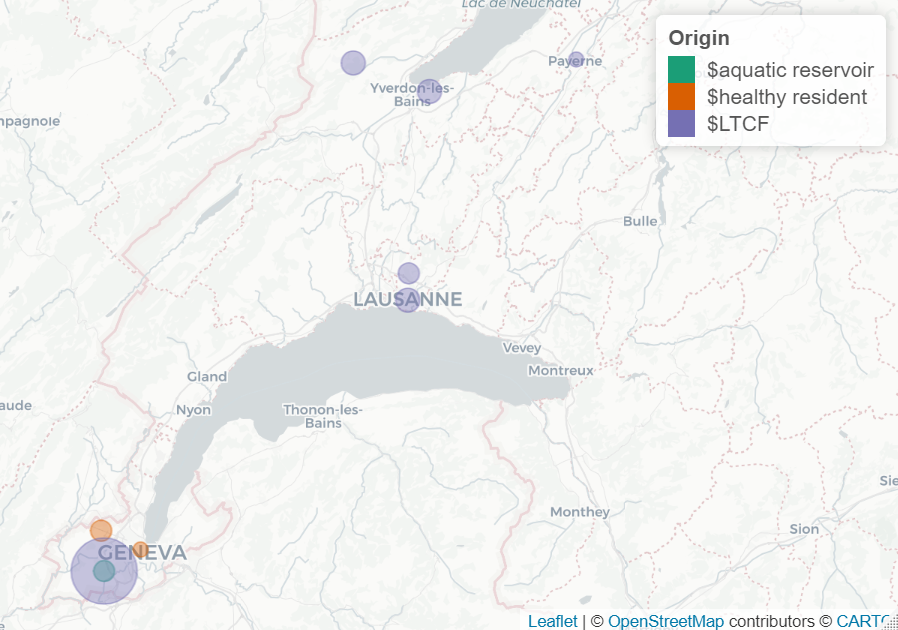


**Figure S2. Minimum Spanning Tree (MST) showing the relationships between all ST131 isolates, analyzed by cgMLST, stratified by sequence type (Key).** ST (Warwick scheme) as defined in ([Wirth, T., Falush, D., **Lan**, R., Colles, F., Mensa, P., Wieler, L.H., Karch, H., Reeves, P. R., Maiden, M. C., Ochman, H., and Achtman M. 2006. Sex and virulence in *Escherichia coli*: an evolutionary perspective. Mol.Microbiol. 60(5), 1136-1151.](http://www.ncbi.nlm.nih.gov/pmc/articles/PMC1557465/)). Shaded links highlight potential clusters (≤10 allele differences, cgmlst.org) of clonally related strains.

**Appendix. References**

1. Decano AG, Downing T. An Escherichia coli ST131 pangenome atlas reveals population structure and evolution across 4,071 isolates. *Sci Rep*. 2019;9(1):17394.

2. Riccio ME, Verschuuren T, Conzelmann N, Martak D, Meunier A, Salamanca E, et al. Household acquisition and transmission of extended-spectrum β-lactamase (ESBL) -producing Enterobacteriaceae after hospital discharge of ESBL-positive index patients. *Clin Microbiol Infect Dis.* 2021;27(9):1322‑9.

3. Martischang R, François P, Cherkaoui A, Gaïa N, Renzi G, Agostinho A, et al. Epidemiology of ESBL-producing Escherichia coli from repeated prevalence studies over 11 years in a long-term-care facility. *Antimicrob Resist Infect Control*. 2021;10(1):148.

4. Kohler P, Seiffert SN, Kessler S, Rettenmund G, Lemmenmeier E, Qalla Widmer L, et al. Molecular Epidemiology and Risk Factors for Extended-Spectrum β-Lactamase-Producing Enterobacterales in Long-Term Care Residents. *J Am Med Dir Assoc*. 2021;S1525-8610(21)00603-4.

5. Martak D, Guther J, Verschuuren TD, Valot B, Conzelmann N, Bunk S, et al. Populations of extended-spectrum β-lactamase-producing Escherichia coli and Klebsiella pneumoniae are different in human-polluted environment and food items: a multicentre European study. *Clin Microbiol Infect Dis*. 2021;S1198-743X(21)00414-6.

6. Wick RR, Judd LM, Gorrie CL, Holt KE. Unicycler: Resolving bacterial genome assemblies from short and long sequencing reads. *PLOS Comput Biol*. 2017;13(6):e1005595.

7. Roer L, Tchesnokova V, Allesøe R, Muradova M, Chattopadhyay S, Ahrenfeldt J, et al. Development of a Web Tool for Escherichia coli Subtyping Based on fimH Alleles. *J Clin Microbiol*. 2017;55(8):2538‑43.

8. Feldgarden M, Brover V, Haft DH, Prasad AB, Slotta DJ, Tolstoy I, et al. Validating the AMRFinder Tool and Resistance Gene Database by Using Antimicrobial Resistance Genotype-Phenotype Correlations in a Collection of Isolates. *Antimicrob Agents Chemother*. 2019;63(11):e00483-19.

9. Seemann T. ABRicate [Internet]. 2022 [cité 17 oct 2022]. Disponible sur: https://github.com/tseemann/abricate

10. Camacho C, Coulouris G, Avagyan V, Ma N, Papadopoulos J, Bealer K, et al. BLAST+: architecture and applications. *BMC Bioinformatics*. 2009;10(1):421.

11. Bortolaia V, Kaas RS, Ruppe E, Roberts MC, Schwarz S, Cattoir V, et al. ResFinder 4.0 for predictions of phenotypes from genotypes*. J Antimicrob Chemother*. 2020;75(12):3491‑500.

12. Zankari E, Allesøe R, Joensen KG, Cavaco LM, Lund O, Aarestrup FM. PointFinder: a novel web tool for WGS-based detection of antimicrobial resistance associated with chromosomal point mutations in bacterial pathogens. *J Antimicrob Chemother*. 2017;72(10):2764‑8.

13. Schürch AC, Arredondo-Alonso S, Willems RJL, Goering RV. Whole genome sequencing options for bacterial strain typing and epidemiologic analysis based on single nucleotide polymorphism versus gene-by-gene–based approaches. *Clin Microbiol Infect*. 2018;24(4):350‑4.
